# The Delivery Strategy of Paclitaxel Nanostructured Lipid Carrier Coated with Platelet Membrane

**DOI:** 10.3390/cancers11060807

**Published:** 2019-06-11

**Authors:** Ki-Hyun Bang, Young-Guk Na, Hyun Wook Huh, Sung-Joo Hwang, Min-Soo Kim, Minki Kim, Hong-Ki Lee, Cheong-Weon Cho

**Affiliations:** 1 College of Pharmacy, Chungnam National University, Daejeon 34134, Korea; robotkr@nate.com (K.-H.B.); youngguk@cnu.ac.kr (Y.-G.N.); hhw3573@nate.com (H.W.H.); zkzkang@naver.com (M.K.); 2 College of Pharmacy and Yonsei Institute of Pharmaceutical Sciences, Yonsei University, 162-1 Songdo-dong, Yeonsu-gu, Incheon 406-840, Korea; sjh11@yonsei.ac.kr; 3 College of Pharmacy, Pusan National University, 63 Busandaehak-ro, Geumjeong-gu, Busan 609-735, Korea; minsookim@pusan.ac.kr

**Keywords:** nanostructured lipid carrier, platelet membrane, biomimicry, paclitaxel

## Abstract

Strategies for the development of anticancer drug delivery systems have undergone a dramatic transformation in the last few decades. Lipid-based drug delivery systems, such as a nanostructured lipid carrier (NLC), are one of the systems emerging to improve the outcomes of tumor treatments. However, NLC can act as an intruder and cause an immune response. To overcome this limitation, biomimicry technology was introduced to decorate the surface of the nanoparticles with various cell membrane proteins. Here, we designed paclitaxel (PT)-loaded nanostructured lipid carrier (PT-NLC) with platelet (PLT) membrane protein because PLT is involved with angiogenesis and interaction of circulating tumor cells. After PLT was isolated from blood using the gravity-gradient method and it was used for coating PT-NLC. Spherical PT-NLC and platelet membrane coated PT-NLC (P-PT-NLC) were successfully fabricated with high encapsulation efficiency (EE) (99.98%) and small particle size (less than 200 nm). The successful coating of PT-NLC with a PLT membrane was confirmed by the identification of CD41 based on transmission electron microscopy (TEM), western blot assay and enzyme-linked immunosorbent assay (ELISA) data. Moreover, the stronger affinity of P-PT-NLC than that of PT-NLC toward tumor cells was observed. In vitro cell study, the PLT coated nanoparticles successfully displayed the anti-tumor effect to SK-OV-3 cells. In summary, the biomimicry carrier system P-PT-NLC has an affinity and targeting ability for tumor cells.

## 1. Introduction

Paclitaxel (PT) is a microtubule inhibitor that promotes polymerization and prohibits dissociation of microtubules. PT has been widely used in the treatment of solid tumors, including breast, ovarian and lung cancers. In particular, PT is the front-line pharmaceutical in ovarian cancer chemotherapy, which is the seventh most common cancer worldwide, with an incidence estimated at around 6.3 per 100,000 women globally [1,2]. Patients with ovarian cancer have a low survival rate [3,4,5]. However, platinum and PT combination chemotherapy is now considered the standard treatment for those with newly diagnosed ovarian cancer, showing excellent response rates ranging from 60% to 70% [6,7].

Despite this, the low solubility of PT and efflux by p-glycoprotein impair its clinical efficacy [8,9,10]. In addition, its use has been limited by significant side effects, including myelosuppression, neutropenia and hypersensitivity [2,11], with neutropenia becoming more profound at higher doses and over longer infusion times. Taxol^®^, the commercial formulation, includes the Cremophor^®^ EL, which caused the side effects. Hypersensitivity is characterized by dyspnea, hypotension and urticaria, all of which are most likely caused by polyoxyl 35 castor oil (Cremophor^®^ EL). During its development, the ortotaxel, PT analogous, showed 50% oral bioavailability, but also had significant, toxic side effects [12]. Its clinical use is limited by reasons of the low bioavailability and adverse effects caused by Cremophor^®^ EL [13].

To overcome these side effects, the development of an alternative drug delivery system for PT is needed. There have been several approaches to developing a PT delivery system, including liposome, emulsion and nanoparticles [9,14,15]. Among these, solid lipid nanoparticles (SLNs) were developed as an alternative drug delivery system (DDS) for liposome emulsion and polymeric nanoparticles [16]. SLNs for antitumor drugs improved the tumor-specific targeting and bioavailability, and showed profound cytotoxicity to multidrug resistant cancer cells [17]. However, the issues such as low drug loading capacity, crystallization of the lipid matrix and drug expulsion have limited the clinical use of SLN.

Nanostructured lipid carrier (NLC) was introduced as an alternative carrier of SLNs [18]. NLC, a type of nanoparticle, is a second-generation lipid nanoparticle composed of both solid and liquid lipids. Using this mixture, NLC has an intense solid state but does not crystallize, allowing higher drug loading [19]. Nanoparticles also possess several limitations to applied clinical use. They may be acted on as a foreign substance, causing an immune response. Although these responses, including immunostimulation and immunosuppression, may be either desirable or undesirable, they may also cause safety concerns [20,21]. Consistent with this, nanoparticles have the potential for elimination by the reticuloendothelial system (RES), which limits the effectiveness of drug delivery to target sites. Further, non-targeted nanoparticles, which exhibit an enhanced permeability and retention (EPR) effect, may cause the unwanted side effect. To overcome these limitations, biomimicry technology was introduced to decorate the surface of the nanoparticles with various cell membrane proteins [22,23].

Platelets (PLTs) are anuclear fragments and small circulating cells in whole blood. They are involved with thrombosis and hemostasis processes, responding to vascular damage and contributing to clot formation. PLTs, also, are involved with angiogenesis and cancer triggers and interact with circulating tumor cells. It has been recognized that the aggregation between PLT and tumor cells correlates with the tumor metastasis. The mechanism of aggregation of platelets surrounding tumor cells includes biomolecular binding such as P-selectin [24] and structure-based capture [25]. Recently, several investigations have shown that tumor-specific targeting can be attained through biomimicry using PLT membrane proteins [22,25,26]. The camouflage using a PLT membrane possesses versatile characteristics. It has been reported that PLT membrane-coated nanoparticles have reduced macrophage cellular uptake and lack particle-induced complement activation [27]. Moreover, the PLT membrane-coated nanoparticles showed a specific affinity to tumor tissues and damaged vasculatures [28].

Therefore, the objective of this study was to fabricate a PT-loaded NLC (PT-NLC) coated with PLTs to overcome the known limitations and increase the anticancer effects.

## 2. Results

### 2.1. Screening of Liquid Lipid, Solid Lipid and Surfactant

The solubility profile of PT in a lipid matrix plays a key role in encapsulation efficiency (EE) and loading capacity (LC) of NLC. In this study, a solubility profile of PT in liquid, solid lipids and surfactant solutions was evaluated.

**Figure 1:**
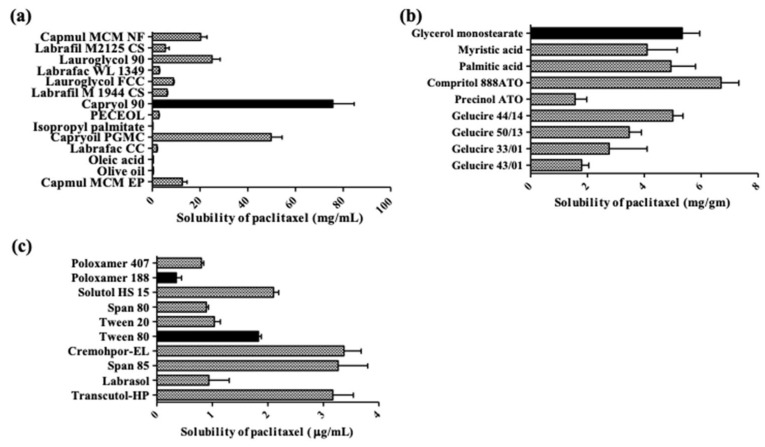
Solubility profiles of paclitaxel (PT) in lipids or surfactants. (**a**), liquid lipids; (**b**), solid lipids; (**c**), 1% surfactant solutions. Black bar represents the liquid, solid lipids and surfactants for PT-nanostructured lipid carrier (NLC).

PT is a lipophilic drug, which have poor water solubility, with a log P value of 3.0. Figure 1a displays the solubility of PT in various liquid lipids. While semisynthetic modified oils (Capryols, Capmuls, Lauroglycols, and Labrafils) showed high solubility, unmodified dietary oils such as oleic acid and olive oil showed low solubility (0.42 ± 0.04 and 0.43 ± 0.06 mg/mL, respectively). In addition, nanoparticles with unmodified dietary oils showed poor emulsification properties. Capryol 90 (propylene glycol monocaprylate) showed the highest solubility (75.72 ± 15.58 mg/mL). High solubility of PT in Capryol 90 might be due to its natural self-emulsifying property [29]; thus, Capryol 90 was selected as the liquid lipid. Figure 1b displays the solubility of PT in various solid lipids. The solubility of PT was high in the following order; Compritol 888 ATO, glyceryl monostearate (GMS) and Gelucire 44/14 (6.7 ± 1.1, 5.3 ± 1.1 and 5.0 ± 0.6 mg/gm, respectively), compared with other solid lipids. The solubility of PT in 1% surfactants solutions is shown in Figure 1c. While tween 80 and poloxamer 188 showed low solubility (3.5 ± 1.6 and 1.0 ± 0.9 μg/mL, respectively), Cremophor^®^ EL, span 85 and transcutol-HP showed high PT solubility (33.70 ± 5.38, 32.66 ± 9.22 and 31.69 ± 0.64 μg/mL, respectively) compared with other surfactant solutions. 

### 2.2. Physicochemical Properties

#### 2.2.1. Particle Size, Polydispersity Index (PDI), Zeta Potential (ZP), EE and LC

The various compositions of formulations of PT-NLC and their physicochemical properties, including particle sizes, PDI, ZP, EE and LC are listed in Table 1a,b, respectively. The final concentration of PT in formulations was fixed at 5 mg/10 mL. For changes in GMS and Capryol 90 ratio (Table 1b), code 4 showed the smallest particle size (115.2 ± 3.9 nm). Code 1 showed a high LC value (3.44 ± 0.01%) and the lowest ZP value (2.24 ± 0.5 mV) compared with the others. ZP is a key factor for characterizing the surface charge of colloids and the stability of the colloidal system. Particles for which the ZP is close to zero tend to be aggregated [30,31]. Based on the ZP values, code 1 was unstable compared with code 4 (−15.00 ± 0.9 mV). In previous studies, nanoparticles with small size tend to accumulate in tumors; this is known as the enhanced permeability and retention (EPR) effect [22,32]. It is expected that small NLCs (particle size < 200 nm) will have increased target specificity for tumor sites. Thus, the fixed ratio of GMS and Capryol 90 (2:1) was used. Next, the total lipid amount was altered and the formulations characterized (Table 1b). The particle size was increased in proportion to the increase in the total lipid amount. However, code 1 and 2 showed the particle size values above 200 nm despite the low volume of total lipid. Thus, the fixed amount of total lipid (210 mg) was used for fabrication of formulation. When the poloxamer 188 or Tween 80 was individually used as a surfactant, the particle size of the formulation was 161.8 or 259.8 nm, respectively. Herein, the code 4 showed the smallest particle size. 

**Figure 2:**
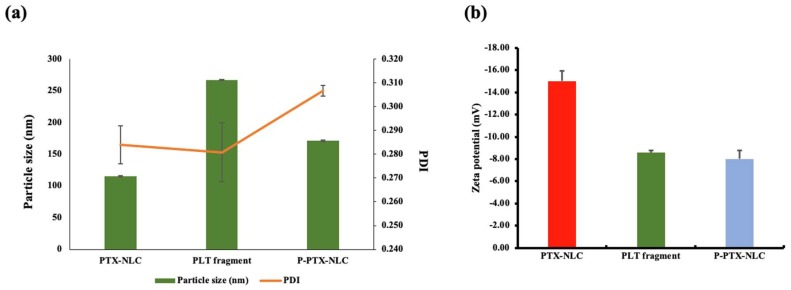
The physicochemical characterizations. (**a**), particle size and polydispersity index (PDI); (**b**), zeta potential of PT-NLC, PLT and P-PT-NLC (*n* = 3, mean ± standard deviation (SD)).

P-PT-NLC was fabricated via a sonication method. The particle size and ZP of P-PT-NLC were 171 ± 0.31 nm and −8.0 ± 0.77 mV, respectively. After the coating of the PLT membrane, the particle size of P-PT-NLC increased compared with that of PT-NLC, but it was smaller than that of PLT fragments (Figure 2a). In addition, when the PLT membrane protein was coated to PT-NLC, ZP decreased to be similar to PLT fragments (Figure 2b). Changes in the particle size and ZP of P-PT-NLC indicated successful coating with PLT membrane [33].

#### 2.2.2. Differential Scanning Calorimetry (DSC) and Powder X-ray Diffraction (PXRD) Analysis

**Figure 3:**
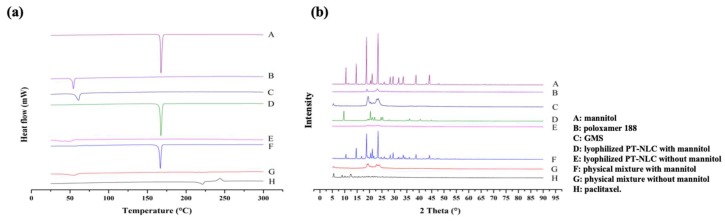
Differential scanning calorimetry (**a**) and powder X-ray diffraction (**b**) analysis.

In general, DSC analysis is used to evaluate the melting behavior or crystallization of nanoparticles. [34,35]. Figure 3a shows the DSC diagram of excipients, PT, lyophilized NLC with or without mannitol and physical mixture with or without mannitol. The melting point of mannitol, poloxamer 188 and GMS were 167 °C, 58 °C and 60 °C, respectively. PT showed two different peaks: endothermal (220 °C) and exothermal (240 °C). In thermograms of PT-NLC formulations, the peak of PT and excipients was decreased. The decreased PT peak of PT-NLC indicates the encapsulation of PT in the lipid matrix [36].

Figure 3b shows PXRD analysis of PT and NLC. PT powder showed a few diffraction peaks at 5.5°, 7.8°, 10.1° and 12.6°. Many crystalline diffraction patterns of PT indicate that PT had crystallinity. Broad peaks were displayed at 19.2° and 24.2° for poloxamer 188, and 19.8° and 24.1° for GMS. These patterns were not displayed in lyophilized NLC without mannitol, but the physical mixture without mannitol showed a PT peak, suggesting that PT was encapsulated in PT-NLC in an amorphous form [37].

#### 2.2.3. Transmission Electron Microscopy (TEM) Analysis

**Figure 4:**
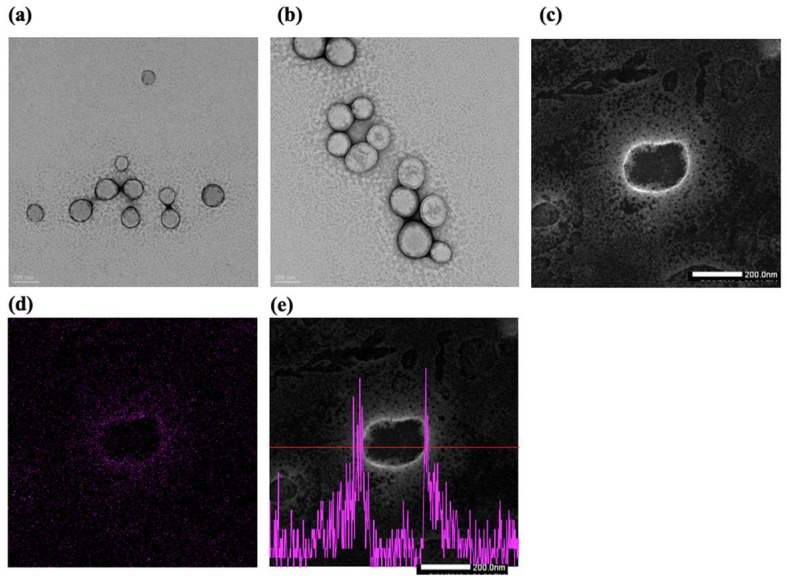
Transmission electron microscopy images of PT-NLC (**a**) and P-PT-NLC (**b**). scanning transmission electron microscopy-energy dispersive X-ray spectroscopy (STEM-EDS) image (**c**), EDS mapping image of uranium elements (**d**) and STEM-EDS line analysis (**e**) of P-PT-NLC.

To confirm the shape and PLT coating on nanoparticles, TEM analysis was conducted with negative staining of uranyl acetate. PT-NLC and P-PT-NLC in Figure 4 show that the nanoparticle morphology of PT-NLC (Figure 4a) and P-PT-NLC (Figure 4b) was spherical.

To evaluate the elemental distribution and composition of P-PT-NLC, EDS mapping and spectra were used (Figure 4). PLT membrane coating was confirmed using scanning transmission electron microscopy-energy dispersive X-ray spectroscopy (STEM-EDS). Figure 4c,d shows the STEM image and EDS mapping of uranium (U) for P-PT-NLC. P-PT-NLC showed a spherical shape with the shell stained by uranyl acetate. In addition, STEM-EDS line analysis showed PLT coating on P-PT-NLC (Figure 4e), indicating that P-PT-NLC was successfully coated with PLT membrane [38,39].

### 2.3. Western Blot Assay and Enzyme-Linked Immunosorbent Assay (ELISA) of CD41

**Figure 5:**
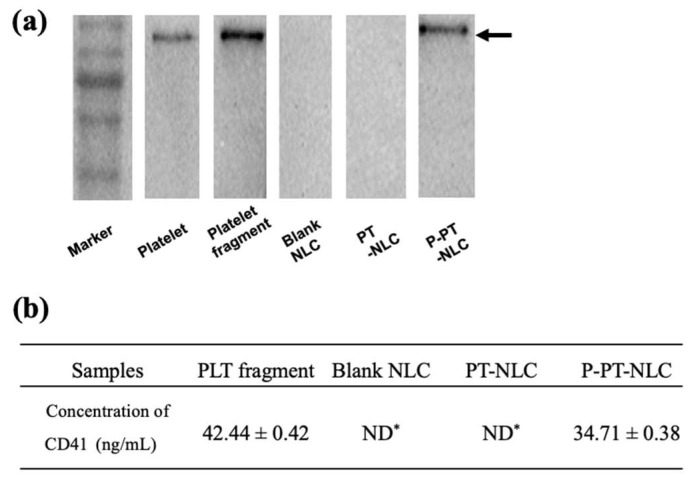
Identification of integrin αIIb (CD41) on P-PT-NLC. (**a**), western blot analysis; (**b**), enzyme-linked immunosorbent assay (ELISA) analysis (*n* = 3, mean ± SD). ND*, not detected. Black arrow indicates the CD41 band (131 kDa).

PLT, PLT fragment, blank-NLC, PT-NLC and P-PT-NLC were separated with 10% polyacrylamide gel stained by coomassie brilliant blue (CBB), and then western blot assay was used to identify the CD41 on P-PT-NLC. CD41, a part of the integrin αIIb, plays a key role in PLT adhesion [40]. CD41 (131 kDa) was detected in PLT, PLT fragment and P-PT-NLC, except for blank-NLC and PT-NLC (Figure 5a, More details can be found in Figure S1), indicating that CD41 protein successfully coated P-PT-NLC.

To quantify CD41 in the formulations, rat PLT membrane glycoprotein 2B3A/CD41^+^CD61^+^ ELISA kit was used. CD41 was detected and quantified in PLT and P-PT-NLC (Figure 5b). The amount of CD41 in P-PT-NLC decreased compared to that in PLT. P-PT-NLC yields using two different analytical methods were calculated and the yields of P-PT-NLC using western blot assay and ELISA were 81.72 ± 0.85 and 79.23 ± 1.08%, respectively.

### 2.4. In Vitro Cell Experiments

**Figure 6:**
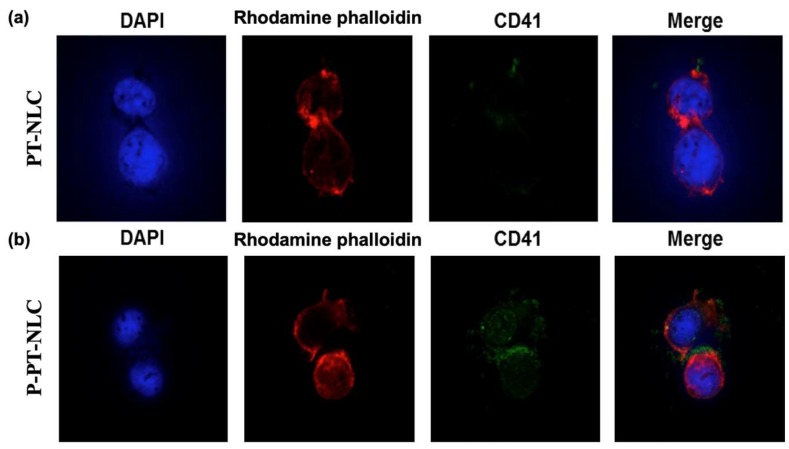
Confocal fluorescent microscopy images. (**a**), PT-NLC; (**b**), P-PT-NLC. After treatment with PT-NLC and P-PT-NLC, SK-OV-3 cell was stained with 4′,6-diamidino-2-phenylindole (DAPI), rhodamine–phalloidin and integrin αIIb Alexa Fluor^®^ 488 antibody (1:100) (blue= nuclear, red = F-actin, green = CD41).

Following this, a confocal laser scanning microscopy (CLSM) study was performed to determine the presence of CD41 on P-PT-NLC. To assess the attachment abilities of nanoparticles to tumor cells, a CLSM study was conducted with PT-NLC and P-PT-NLC on SK-OV-3 cells. CLSM images of PT-NLC and P-PT-NLC are shown in Figure 6. In Figure 6b, CD41 on the surface of P-PT-NLC was detected on SK-OV-3 cells. By contrast, CD41 was not detected in PT-NLC (Figure 6a). In merging the images of P-PT-NLC, CD41 (green fluorescence) was co-localized in 4′,6-diamidino-2-phenylindole (DAPI) (blue fluorescence) and rhodamine–phalloidin (red fluorescence) staining sites. This indicates that P-PT-NLC was attached to SK-OV-3 cancer cells and that CD41 on P-PT-NLC possesses a high tumor-cell affinity [33,40,41].

**Figure 7:**
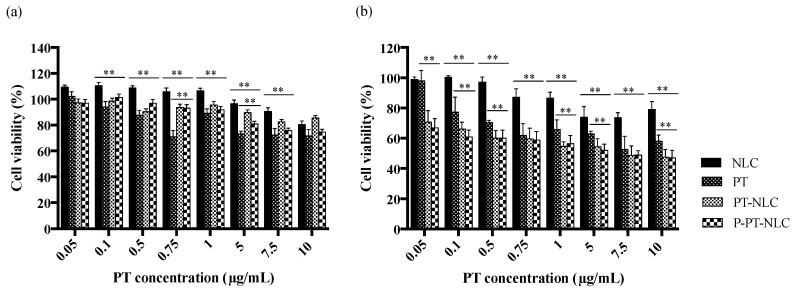
Cytotoxicity assay of PT, PT-NLC and P-PT-NLC in SK-OV-3 cells after 24 h (**a**) and 48 h (**b**). ** *p* < 0.01 (multiple *t*-tests).

The cytotoxic effects of PT, blank-NLC, PT-NLC and P-PT-NLC at 24 and 48 h were evaluated with SK-OV-3 cells (Figure 7). Blank-NLC consisted of an equivalent lipid concentration of PT-NLC and P-PT-NLC. In the 0.1–7.5 μg/mL concentration range, all formulations were more toxic compared with blank-NLC after 24 h. The cell viability of blank-NLC at 10 μg/mL was 71.56 ± 10.12%; therefore, the excipient of NLC showed cytotoxicity to SK-OV-3 cells (Figure 7a). At 10 μg/mL, the cell viability of PT (, PT-NLC (47.52 ± 5.10%) and P-PT-NLC (47.07 ± 4.95%) were less than that of blank-NLC (79.07 ± 5.24%) at 48 h post-incubation. PT-NLC and P-PT-NLC were significantly more toxic than PT (*p* < 0.01) at all concentration ranges, but no significant difference was observed between PT-NLC and P-PT-NLC (Figure 7b), indicating that the release profile of PT-NLC and P-PT-NLC was a sustained release.

### 2.5. Biodistribution Study

**Figure 8:**
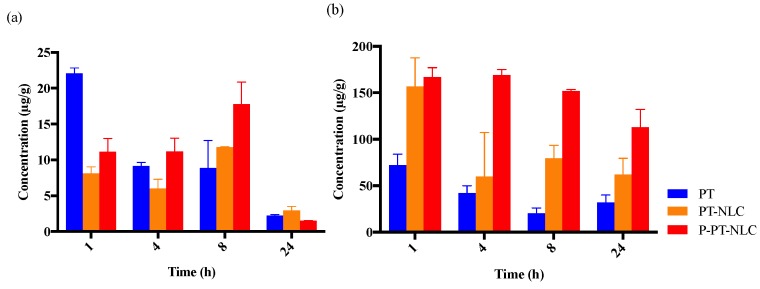
Biodistribution at the 10 mg/kg dose of PT, PT-NLC and P-PT-NLC in kidney (**a**) and liver (**b**) at 1, 4, 8 and 24 h. Each column represents the mean ± SD (*n* = 2).

Following administration of PT, PT-NLC and P-PT-NLC, PT concentration was continuously eliminated in the kidney. However, in PT-NLC and P-PT-NLC, PT concentration increased to 8 h and was eliminated at 24 h; therefore, it seems to accumulate in the kidney, while the PT concentration was reduced at 24 h (Figure 8a). This indicates that PT-NLC and P-PT-NLC were sufficiently eliminated from the kidney. PT-NLC and P-PT-NLC accumulated in the liver at higher levels compared to PT (Figure 8b). This issue could be explained by the reticuloendothelial system (RES) or liver Kupffer cells uptake [9,42]. When particle size is between 50 and 200 nm, nanoparticles tend to accumulate in the liver by RES. This indicates that nanoparticles with a diameter of 50–200 nm were caught by the RES, resulting in sustained release [23,43]. PT-NLC and P-PT-NLC tended to accumulate in the liver more than PT, but PT-NLC and P-PT-NLC appeared to be eliminated from the liver over time. This means that as the nanoparticles are taken up by the RES, they are slowly released over time. Moreover, it has been reported that the liver was one of the major organs for the physiological clearance of PLT [44]. To clarify this issue, additional liver toxicity studies should be conducted for PT-NLC and P-PT-NLC.

## 3. Discussion

In this study, PT-NLC was fabricated considering the EE and the particle size. Composition of PT NLC with the highest EE value and the optimal particle size (less 200 nm) [45] was selected for the fabrication of P-PT-NLC. Compritol 888ATO showed the highest solubility as a solid lipid; however, PT-NLC with Compritol 888ATO showed the excessive solidification during the fabrication. Thus, GMS was selected as the solid lipid. Surfactant, which has low drug solubility, affects the association between the drug and lipid matrix and materializes drugs in the lipid core. Surfactant use with NLC leads to drug distribution in the core rather than the exterior of the NLC [29,46]. Thus, non-ionic surfactant provides the steric hindrance and static repulsion for particles [32]. Previously, we successfully fabricated the NLC-loaded ticagrelor using the poloxamer 188 and Tween 80 as surfactants [47]. In addition, the previous study suggested that using Poloxamer 188 (1%), a steric stabilizer, with Tween 80 (2%) as a surfactant, in the manufacture of NLC could provide a stable NLC with a particle size below 100 nm [48]. Thus, Tween 80 and poloxamer 188 were selected as surfactant and co-surfactant with a ratio of 2:1, respectively.

To confirm whether PT was encapsulated in NLC, DSC and PXRD patterns were conducted with NLC and its ingredients (Figure 3). When PT-NLC was freeze-dried with the lyophilizing agent, mannitol, the PT peak decreased. However, the mannitol peak was still high; therefore, blank NLC and PT-NLC were lyophilized without mannitol to remove its effect on the DSC and PXRD patterns. The PT peak was decreased in freeze-dried PT-NLC. However, the physical mixture still had a PT peak, indicating that the crystallites of PT were not detected in lyophilized PT-NLC without mannitol and that PT was encapsulated successfully in the lipid matrix.

When P-PT-NLC was fabricated with PT-NLC and PLT by sonication, particle size increased by around 50 nm and ZP was similar to PLT fragment. It is reported that the size of nanoparticles coated with PLT was about 20 nm larger than the bare nanoparticles in previous studies [22,49,50]. Moreover, it is stated that the ZP of nanoparticles coated with PLT was similar to that of the surface charge of PLT in accordance with the previous investigation. P-PT-NLC with increased size and charged to the same level as a PLT fragment means that P-PT-NLC was coated with PLT.

The shapes of PT-NLC and P-PT-NLC were inspected and confirmed by TEM (Figure 4). Nanoparticles were spherical and the particle size was similar compared with electrophoretic light scattering (ELS) data. In addition, PT-NLC and P-PT-NLC were negatively stained with uranyl acetate, which can bind to surfaces of the phosphate moiety of lipid structures by ionic interaction [51]. The PLT membrane was composed of phospholipid, so uranyl acetate could interact with the PLT membrane. Using this interaction between the PLT membrane and uranyl acetate, STEM/EDS analysis was utilized to confirm the PLT membrane coating in P-PT-NLC. In the STEM image (Figure 4c), PLT membrane on P-PT-NLC was shown as a white line from uranyl acetate, indicating that uranium may be present on the PLT membrane. The intensity of uranium was investigated using EDS mapping (Figure 4d) and line analysis (Figure 4e), and PLT coating in P-PT-NLC was confirmed, with strong uranium intensity, on the surface of P-PT-NLC. 

Before the western blot assay, sodium dodecyl sulfate–polyacrylamide gel electrophoresis (SDS-PAGE) was used to separate various proteins from PLT fragments and to identify PLT membranes in P-PT-NLC. The SDS-PAGE patterns of PLT and PLT fragments were consistent with that reported in a previous study [30]. In the western blot assay, the PLT marker (CD41) was detected in P-PT-NLC, suggesting that PT-NLC was successfully coated with the PLT fragment. In summary, the PLT membrane proteins were successfully translocated to PT-NLC. However, PLT pattern intensity in P-PT-NLC was lower compared with PLT and PLT fragments, possibly due to the purification of P-PT-NLC. Because of this step, PLT pattern intensity was decreased compared with PLT fragment. Similar result was obtained by ELISA.

The affinity of P-PT-NLC for SK-OV-3 cells was confirmed by CLSM (Figure 6). It has been reported that PLTs play a role in the survival, migration and growth of tumor cells [52,53]. In fact, anticoagulants have been shown to reduce tumor growth, metastasis and angiogenesis [54,55]. In this experiment, CD41 was used as a PLT maker. When P-PT-NLC was treated with SK-OV-3 cells, CD41 was observed on the surface of SK-OV-3 cells. However, no CD41 was detected with PT-NLC. CD41 was co-localized on the cytoplasm rather than the nuclei, indicating that P-PT-NLC was bound to the surface of tumor cells and that PLT coated particles might be used to target tumor cells.

Cytotoxicity to ovarian cancer cells was confirmed by MTT assay (Figure 7). Blank NLC did not show cytotoxicity at all concentrations because cell viability exceeded 80%. However, PT, PT-NLC and P-PT-NLC showed cytotoxicity to SK-OV-3 cells. After 48 h of incubation, low doses of PT-NLC and P-PT-NLC produced cytotoxic effects, while PT did not. In addition, the cytotoxic effects of PT-NLC and P-PT-NLC were superior compared with PT, suggesting that PT-NLC and P-PT-NLC produce a profound in vitro cytotoxic effect in ovarian cancer cells.

An in vivo biodistribution study of PTC-NLC and P-PT-NLC showed late elimination from the kidney and liver (Figure 8). In the kidney, nanoparticles seemed to accumulate at an early stage after i.v. injection. However, similar amounts of nanoparticles and PT remained in kidney at 24 h. The nanoparticles coated with PLT were found to be distributed to the kidney at the similar level of the bare nanoparticles after 24 h administrations, as has previously been investigated [50]. In the liver, however, formulations accumulated up to 24 h after i.v. injection. It may be that nanoparticles accumulate by RES in the liver causing hepatoxicity. However, nanoparticles were also excreted from the liver over the study period, indicating that nanoparticles were slowly released into systemic circulation. Therefore, hepatotoxicity analyses of PT-NLC and P-PT-NLC are needed to determine liver toxicity.

## 4. Materials and Methods 

### 4.1. Chemicals and Reagents

PT and docetaxel (internal standard, IS) were gifted by Korea United Pharm, Inc. (Seoul, Korea). Isopropyl palmitate, palmitic, myristic and stearic acid were purchased from Daejung Chemical (Cheongwon, Korea). Tween 80, Tween 20, GMS, oleic acid, Span 85 and 80, phosphoric acid and mannitol were obtained Samchun Chemical (Pyungtaek, Korea). Capmul^®^ MCM-NF and -EP were purchased from Abitec Corporation (Columbus, OH, USA). Gelucire 44/14, 33/01, 43/01, 50/13, Capryol™ PGMC, Compritol^®^ 888 ATO, Capryol 90, Labrafac™ WL 1349 and CC, Labrasol, Cremophor^®^ EL, Labrafil^®^ M 2125 CS, Peceol, Lauroglycol 90, Transcutol^®^ HP, Lauroglycol™ FCC, precirol ATO, and Labrafil^®^ M 1944 CS were obtained by Gattefossé (Saint Priest, Cedex, France). Poloxamer 188, 407 and Solutol^®^ HS-15 were obtained from BASF (Ludwigshafen, Germany). Dimethyl sulfoxide (DMSO), DAPI, 3-(4,5-dimethylthoazol-2yl)-2,5-diphenyl-2H-tetrazolium bromide (MTT) and rhodamine–phalloidin were obtained from Sigma-Aldrich (St. Louis, MO, USA). SK-OV-3 cells, an ovarian cancer cell line, were obtained from the Korean Cell Line Bank (Seoul, Korea). High-performance liquid chromatography (HPLC)-grade methanol (MeOH) and acetonitrile (ACN) were obtained from JT Baker (Phillipsburg, NJ, USA).

### 4.2. Screening of Liquid Lipid, Solid Lipid and Surfactant

PT solubility in liquid lipids, solid lipids and surfactant solutions was evaluated for screening of liquid, solid lipid and surfactant. To evaluate the PT solubility in solid lipids, briefly, 1 g of various solid lipids was weighed to glass vials and boiled in a water. PT was added until the saturation of PT in the solid lipid. The amount of PT dissolved in solid lipids was considered as its solid lipid solubility. To evaluate the PT solubilities in liquid lipids and surfactants, excess amount of PT was added to 0.5 mL of various oils and 1% (*w*/*v*) surfactants and mixed at 1000 rpm for 72 h. After the centrifugation (15,000× *g* for 10 min), the supernatant was diluted with ACN and PT solubility was evaluated using a HPLC with an ultraviolet (UV) detector. All samples were performed in triplicate.

### 4.3. PT-NLC Preparation

PT-NLC was fabricated using the hot melt emulsification and sonication methods. In each experiment, liquid (Capryol 90^®^) and solid lipid (GMS) were mixed in a 75 °C water bath. Five milligrams of PT were added to the melted lipids and mixed. Heated Tween 80 and poloxamer 188 were added and homogenized at 15,000 rpm for 1 min. They were sonicated with 108 W of amplitude for 10 min. After the cooling, formulations were freeze-dried with or without mannitol by lyophilizer (FD-1000, EYELA, Tokyo, Japan). Blank-NLC was fabricated without PT.

### 4.4. P-PT-NLC Preparation

PLTs were isolated using the gravity-gradient method as previously described [26], with slight modification. Briefly, whole blood was collected in anticoagulated tubes with sodium citrate from rat jugular veins. PLTs with the number of 10^8^–10^9^ cell/mL were used and the number of PLTs was measured by an automated blood counter. Whole blood samples were centrifuged at 200 *g* for 10 min and supernatant was collected, and this was centrifuged at 110× *g* for 6 min to discard red and white blood cells, after which the supernatants were collected in a tube. This was centrifuged at 1500× *g* for 15 min to separate the PLT pellet. Pellet was washed three times with phosphate-buffered saline (PBS) containing protease inhibitor (Thermo Scientific, Waltham, MA, USA). The final number of PLT was 10^3^–10^4^ cell/mL for P-PT-NLC fabrication.

A preselected PT-NLC formulation was used to prepare P-PT-NLC. Two hundred microliters of PLT concentrate were added to 10 mL of PT-NLC, which was then sonicated using Vibra-Cell (amplitude 80 W, 3 min, turned on for 5 s and off for 2 s per cycle). P-PT-NLC was then placed in an ice bath for cooling and used for the characterization study.

### 4.5. Physicochemical Properties of Formulations

#### 4.5.1. Evaluation of Particle Size, PDI and ZP

Various formulations (code 1–8) were prepared by increasing the solid lipid (GMS) amount in fixing liquid lipid, increasing the total lipid amount in fixing the ratio between solid lipid and liquid lipid, and changing the surfactant ratio.

Physiochemical parameters, including the particle sizes and PDIs of various PT-NLC formulations were measured using a laser scattering analyzer (ELS-8000, Otsuka Electronics, Osaka, Japan). ZPs of different PT-NLC nanoparticles were assessed using a Zetasizer Nano Z (Malvern Panalytical Ltd., Malvern, UK). 

#### 4.5.2. DSC and PXRD Analysis

To evaluate the thermal characteristics of formulations and change of crystallinity of ingredients in PT-NLC, DSC and PXRD analysis were performed using a DSC N-650 thermal analyzer (Scinco, Seoul, Korea) and D/Max-2200 Ultima/PC (Rigaku, Tokyo, Japan) with Ni filtered Cu-Kα (40 kV, 40 mA). For DSC analysis, briefly, samples were heated from 25 °C to 300 °C at a heating rate of 10 °C/min using an aluminum pan under nitrogen gas flow. PXRD analysis was conducted from 5° to 60° with a step size of 0.02 °/s.

#### 4.5.3. TEM Analysis

Morphology of PT-NLC and P-PT-NLC were evaluated using a JEM 2100F field emission electron microscope (JEOL Ltd., Tokyo, Japan) at 200 kV. In addition, STEM-EDS analysis was performed. Samples were placed on a 200 mesh copper grid and washed with distilled water. Then, they were negatively stained with 2% uranyl acetate (Agar Scientific Ltd., Stansted, UK).

#### 4.5.4. EE and LC Determination

The PT content of samples was diluted with ACN and determined by HPLC. Sample EE and LC were evaluated using an ultrafiltration method. Two hundred microliters of sample were added to the chambers of the centrifuge tube in an Amicon filter (10 kDA, Millipore, Billerica, MA, USA), which was centrifuged at 14,000× *g* for 30 min at 4 °C. The pellet was diluted with ACN to measure the free PT of formulations by the HPLC system. EE and LC were determined as follows:

(1) $$\mathrm{EE}\;\left(\%\right)\;=\;\frac{\mathrm{Total}\;\mathrm{amount}\;\mathrm{of}\;\mathrm{PT}\;-\;\mathrm{Amount}\;\mathrm{of}\;\mathrm{free}\;\mathrm{PT}}{\mathrm{Total}\;\mathrm{amount}\;\mathrm{of}\;\mathrm{PT}}\;\times \;100$$

(2) $$\mathrm{LC}\;\left(\%\right)\;=\;\frac{\mathrm{Total}\;\mathrm{amount}\;\mathrm{of}\;\mathrm{PT}\;\mathrm{in}\;\mathrm{NLC}}{\mathrm{Total}\;\mathrm{amount}\;\mathrm{of}\;\mathrm{lipids}\;+\;\mathrm{Total}\;\mathrm{amount}\;\mathrm{of}\;\mathrm{PT}\;\mathrm{in}\;\mathrm{NLC}}\;\times \;100$$

### 4.6. Characterization of PLT Membrane Protein Coated NLC (P-PT-NLC)

To evaluate the PLT coating, PLT membrane protein integrin αIIb (CD41) was identified and quantified using electrophoresis, western blot and enzyme-linked immunosorbent assay (ELISA). For all methods, the degree of PLT coating was assessed as relative intensity:

(3) $$\mathrm{Relative}\;\mathrm{intensity}\;\left(\%\right)\;=\;\frac{\mathrm{Intensity}\;\mathrm{of}\;P-\mathrm{PT}-\mathrm{NLC}}{\mathrm{Intensity}\;\mathrm{of}\;\mathrm{PLT}}\;\times \;100$$

#### 4.6.1. Western Blot

Samples were added in loading buffer (Biosesang Inc., Sungnam, Korea) and heated to 100 °C for 10 min. Samples were loaded on 10% polyacrylamide gel and separated at 100 V for 120 min. Samples on gels were transferred to a polyvinylidene difluoride (PVDF) membrane at 80 V for 180 min. Membranes were blocked with 5% bovine serum albumin at room temperature for 1 h. Then, membranes were incubated with integrin αIIb horseradish peroxidase (HRP) antibody (1:1000 dilution; Santa Cruz Biotechnology, Inc., Dallas, TX, USA) at room temperature overnight. They were treated with ECL^®^ prime western blotting detection reagent (GE Healthcare Bio-Sciences, Pittsburgh, PA, USA). CD41 (131k Da) band was detected using the Fusion SL2 chemiluminescent imaging system (Vilber Lourmat, Marne-la-Vallée Cedex, France). P-PT-NLC yield was measured by comparing the CD41 band intensity of PLT fragment and P-PT-NLC.

#### 4.6.2. ELISA

To quantify CD41, rat PLT membrane glycoprotein 2B3A/CD41+CD61+ELISA kit was used (MyBioSource, Inc., San Diego, CA, USA). Briefly, 50 µL of sample were placed in each well and 100 µL of HRP-conjugate reagent were added. After the incubation at 37 °C for 60 min under dark conditions, plate was washed three times with wash solution. Each 50 µL of chromogen solution A and B was added to each well. The plate was incubated under dark conditions at 37 °C for 15 min. After the addition of stop solution, the optical density was measured using a microplate reader (Sunrise, Tecan, Männedorf, Switzerland) at 450 nm within 15 min. P-PT-NLC yield was measured by comparing the CD41 value of PLT fragment and P-PT-NLC.

#### 4.6.3. CLSM Analysis

SK-OV-3 (10^6^ cell) was preincubated overnight and samples was added to the cells. Integrin αIIb Alexa Fluor^®^ 488 antibody (1:100 dilution; Santa Cruz Biotechnology, Dallas, TX, USA) was treated for 3 h. Then, cells were washed three times with PBS and stained with DAPI and rhodamine–phalloidin. Fluorescence images were analyzed using a super resolution confocal laser scanning microscope (LSM880 with Airyscan; Carl Zeiss, Oberkochen, Germany)

### 4.7. Cytotoxicity Study

Human ovarian cancer cell line SK-OV-3 was cultured with Dulbecco’s modified eagle medium (DMEM) supplemented with 10% fetal bovine serum (FBS), 100 µg/mL of streptomycin and 100 U/mL of penicillin at 37 °C in a humidified incubator supplied with 5% CO_2_ atmosphere.

The cytotoxicity of formulations against SK-OV-3 cells was determined using MTT assay. Cells (5 × 10^4^ cells/well) were prepared and various concentrations of PT, blank-NLC, PT-NLC and P-PT-NLC (0.01–10 µg/mL) were added to cells. Blank-NLC consisted of an equivalent lipid concentration of PT-NLC and P-PT-NLC. At 24 and 48 h post-incubation, 30 µL of MTT solution (5 mg/mL) was added and the plate was incubated for 3 h. Mediums on the plate were discarded and DMSO was added to each well. The absorbance was measured with a microplate reader (Sunrise, Tecan, Austria) at 560 nm. Cell viability was calculated as:

(4) $$\mathrm{Cell}\;\mathrm{viability}\;(\%)\;=\;\frac{{\mathrm{OD}}_{\mathrm{test}}}{{\mathrm{OD}}_{\mathrm{control}}}\times \;100$$

where OD_test_ is the absorbance of the cells treated with PT and OD_control_ is the absorbance intensity of the cells incubated with 0.1% DMSO in DMEM medium.

### 4.8. Biodistribution Study

Male Sprague–Dawley rats (age: 7–8 weeks; body weight: 400–420 g) were provided by Samtako (Osan, Korea) for the biodistribution study. Rats were housed at 22 °C with a 12-h light–dark cycle and given free access to food and water. The rats were acclimated for 1 week prior to the experiments. All procedures were approved by the Local Ethical Committee of Chungnam National University (Protocol No. CNU-00911).

Animals were randomly divided into three groups (8 animals for each group): group A (PT), group B (PT-NLC) or group C (P-PT-NLC). Before the injection of formulations, animals were fasted for 12 h. PT as a control was dissolved in Cremophor^®^ EL and ethanol (1:1, *w*/*w*) and diluted with normal saline. Freeze-dried PT-NLC and P-PT-NLC were prepared to dilute with normal saline. Animals in groups A, B and C received PT, PT-NLC or P-PT-NLC, respectively, via IV to the tail vein at a dose of 10 mg/kg. After the injection, animals were killed by CO_2_ at 1, 4, 8 or 24 h (2 animals for each time point), and organs (kidneys and livers) were collected.

For the preparations of samples, 2 mL of 0.15 M NaCl solution and 2 g of kidney were homogenized at 15,000 rpm for 10 min using a homogenizer. For liver samples, 5 g of liver and 5 mL of distilled water were homogenized at 15,000 rpm for 10 min. Then, 25 µL of IS solution (5 µg/mL of docetaxel in ACN) was added to 100 µL of tissue sample. After the addition of 1 mL ACN, samples were vortexed and shaken for 2 min. Samples were centrifuged for 10 min at 15,000× *g* and 900 µL of organic layer was collected and evaporated with nitrogen gas. Evaporated samples were reconstituted with 50 µL of distilled water and ACN mixture (1:1). After the centrifugation (15,000× *g* for 10 min), 40 µL of supernatant was injected into the HPLC system.

### 4.9. HPLC Condition

Agilent 1100 HPLC system (Santa Clara, CA, USA) with a UV detector was used for HPLC analysis of PT. Kinetex^®^ C18 column (4.6 × 250 mm, 100 Å, 5 µm; Phenomenex, Torrance, CA, USA) was used and the mobile phase was consisted of ACN and 0.1% phosphoric acid buffer (45:55%, *v*/*v*). The flow rate and column temperature were 1.3 mL/min and 30 °C. The injection volume was 20 µL and UV detection of PT was performed at 227 nm.

For the biodistribution study, the mobile phase of ACN and 0.1% phosphoric acid buffer (55:45%, *v*/*v*) was used and the injection volume was 40 µL.

### 4.10. Data and Statistical Analysis

Data was evaluated using a *t*-test to determine the significant differences among groups. Data was expressed as mean ± standard deviation (SD). All analysis was conducted using a GraphPad Prism (Graph-Pad Software, CA, USA). Western blot data were evaluated using Image J software (National Institutes of Health, Bethesda, MD, USA).

## 5. Conclusions

Herein, the P-PT-NLC was fabricated to avoid an immune response and target tumor cells. PT-NLC was successfully fabricated using hot melt emulsification and sonication methods. In addition, the PLT membrane protein was successfully coated using a sonication method. Spherical PT-NLC and P-PT-NLC were fabricated with high EE (99.98%) and small particle size (less than 200 nm). Based on TEM, western blot assay and ELISA data, the P-PT-NLC was enclosed by CD41, a PLT membrane protein. Moreover, we confirmed the affinity of P-PT-NLC for tumor cells. PT-NLC and P-PT-NLC showed the cytotoxic effect to SK-OV-3 cells. In a biodistribution study, NLC formulation was distributed to both the liver and kidney. In summary, P-PT-NLC has an affinity and targeting ability for tumor cells. This might be due to PLTs, which play a key role in various physiologic and pathologic processes. Biomimicry carrier systems, especially PLT membrane coating, promise new drug delivery platforms.

## Figures and Tables

**Table 1:** Various compositions for PT-NLCs (a) and their physicochemical properties (b) (*n* = 3).

**(a) Various Compositions for PT-NLCs**					
**Code**	**GMS (mg)**	**Capryol 90 (mg)**	**PT (mg)**	**% of Poloxamer 188 in 10 mL**	**% of Tween 80 in 10 mL**
1	70	70	5	0.5	1
2	120	60	5	0.5	1
3	140	70	5	1	0.5
4	140	70	5	0.5	1
5	160	80	5	0.5	1
6	180	90	5	0.5	1
7	210	70	5	0.5	1
8	280	70	5	0.5	1
**(b) Physicochemical Properties**					
**Code**	**Particle Size (nm)**	**PDI**	**EE (%)**	**LC (%)**	**ZP (mV)**
1	279.2 ± 10.4	0.308 ± 0.012	99.79 ± 0.18	3.44 ± 0.01	2.24 ± 0.51
2	266.5 ± 46.8	0.360 ± 0.011	99.94 ± 0.03	2.70 ± 0.03	−22.70 ± 1.46
3	161.3 ± 0.9	0.311 ± 0.301	99.62 ± 0.05	2.25 ± 0.01	−16.40 ± 0.82
4	115.2 ± 3.9	0.284 ± 0.015	99.98 ± 0.01	2.33 ± 0.01	−15.00 ± 0.93
5	123.0 ± 0.9	0.352 ± 0.022	99.95 ± 0.02	2.04 ± 0.01	−30.60 ± 0.31
6	164.4 ± 12.2	0.321 ± 0.017	99.94 ± 0.05	1.82 ± 0.02	−29.40 ± 1.59
7	158.6 ± 9.8	0.297 ± 0.012	99.96 ± 0.06	1.75 ± 0.01	−25.33 ± 0.62
8	2599.4 ± 392.7	0.220 ± 0.150	99.51 ± 0.03	1.40 ± 0.01	−31.43 ± 0.54

## Supplementary Materials

The following are available online at https://www.mdpi.com/2072-6694/11/6/807/s1, Figure S1: The whole membrane with all molecular weight markers on the western blot.
