# Supplementary material for: The Delivery Strategy of Paclitaxel Nanostructured Lipid Carrier Coated with Platelet Membrane
Source: Cancers (Basel). 2019 Jun 11;11(6):807. doi: 10.3390/cancers11060807 (PMC6627627; doi:10.3390/cancers11060807)
Supplement: Supplementary file 1 [file cancers-11-00807-s001.pdf]

Supplementary Materials: The Delivery Strategy of Paclitaxel Nanostructured Lipid Carrier Coated with Platelet Membrane

Ki-Hyun Bang, Young-Guk Na, Hyun Wook Huh, Sung-Joo Hwang, Min-Soo Kim, Minki Kim, Hong-Ki Lee and Cheong-Weon Cho

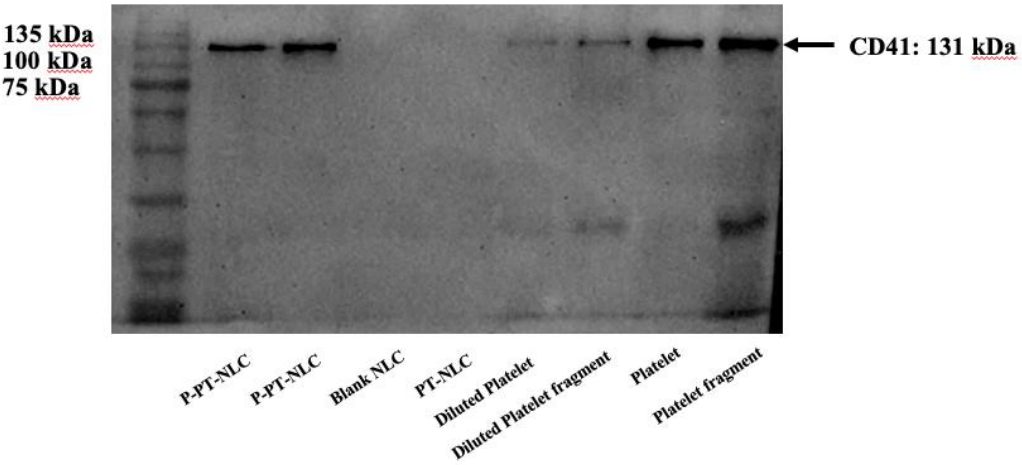

Figure S1. The whole membrane with all molecular weight markers on the western blot.

© 2019 by the authors. Licensee MDPI, Basel, Switzerland. This article is an open access article distributed under the terms and conditions of the Creative Commons Attribution (CC BY) license (http://creativecommons.org/licenses/by/4.0/).
